# Supplementary material for: Meiosis-specific ZFP541 repressor complex promotes developmental progression of meiotic prophase towards completion during mouse spermatogenesis
Source: Nat Commun. 2021 Jun 1;12:3184. doi: 10.1038/s41467-021-23378-4 (PMC8169937; doi:10.1038/s41467-021-23378-4)
Supplement: Supplementary file 1 — Supplementary information [file 41467_2021_23378_MOESM1_ESM.pdf]

Supplementary information

Meiosis-specific ZFP541 repressor complex promotes developmental progression of meiotic prophase towards completion during spermatogenesis

Yuki Horisawa-Takada[1], Chisato Kodera[1, 8, 11], Kazumasa Takemoto[1, 11], Akihiko Sakashita[2], Kenichi Horisawa[3], Ryo Maeda[4], Ryuki Shimada[1], Shingo Usuki[5], Sayoko Fujimura[5], Naoki Tani[5], Kumi Matsuura[6], Tomohiko Akiyama[7], Atsushi Suzuki[3], Hitoshi Niwa[6], Makoto Tachibana[4], Takashi Ohba[8], Hidetaka Katabuchi[8], Satoshi H. Namekawa[9], Kimi Araki[10], and Kei-ichiro Ishiguro[1]*

1 Department of Chromosome Biology, Institute of Molecular Embryology and Genetics (IMEG), Kumamoto University, Kumamoto, 860-0811 Japan
2 Department of Molecular Biology, Keio University School of Medicine, Tokyo160-8582, Japan
3 Division of Organogenesis and Regeneration, Medical Institute of Bioregulation, Kyushu University, Fukuoka 812-8582, Japan
4 Graduate School of Frontier Biosciences, Osaka University, Osaka 565-0871 Japan
5 Liaison Laboratory Research Promotion Center, IMEG, Kumamoto University, 860-0811 Japan
6 Department of Pluripotent Stem Cell Biology, IMEG, Kumamoto University, 860-0811 Japan
7 Department of Systems Medicine, Keio University School of Medicine, Tokyo 160-8582, Japan
8 Department of Obstetrics and Gynecology, Faculty of Life Sciences, Kumamoto University, 860-8556 Japan
9 Department of Microbiology and Molecular Genetics, University of California, Davis CA95616, USA
10 Institute of Resource Development and Analysis, and Center for Metabolic Regulation of Healthy Aging, Kumamoto University, Kumamoto, 860-0811 Japan
11 equally contributed to this work
* correspondence : ishiguro@kumamoto-u.ac.jp

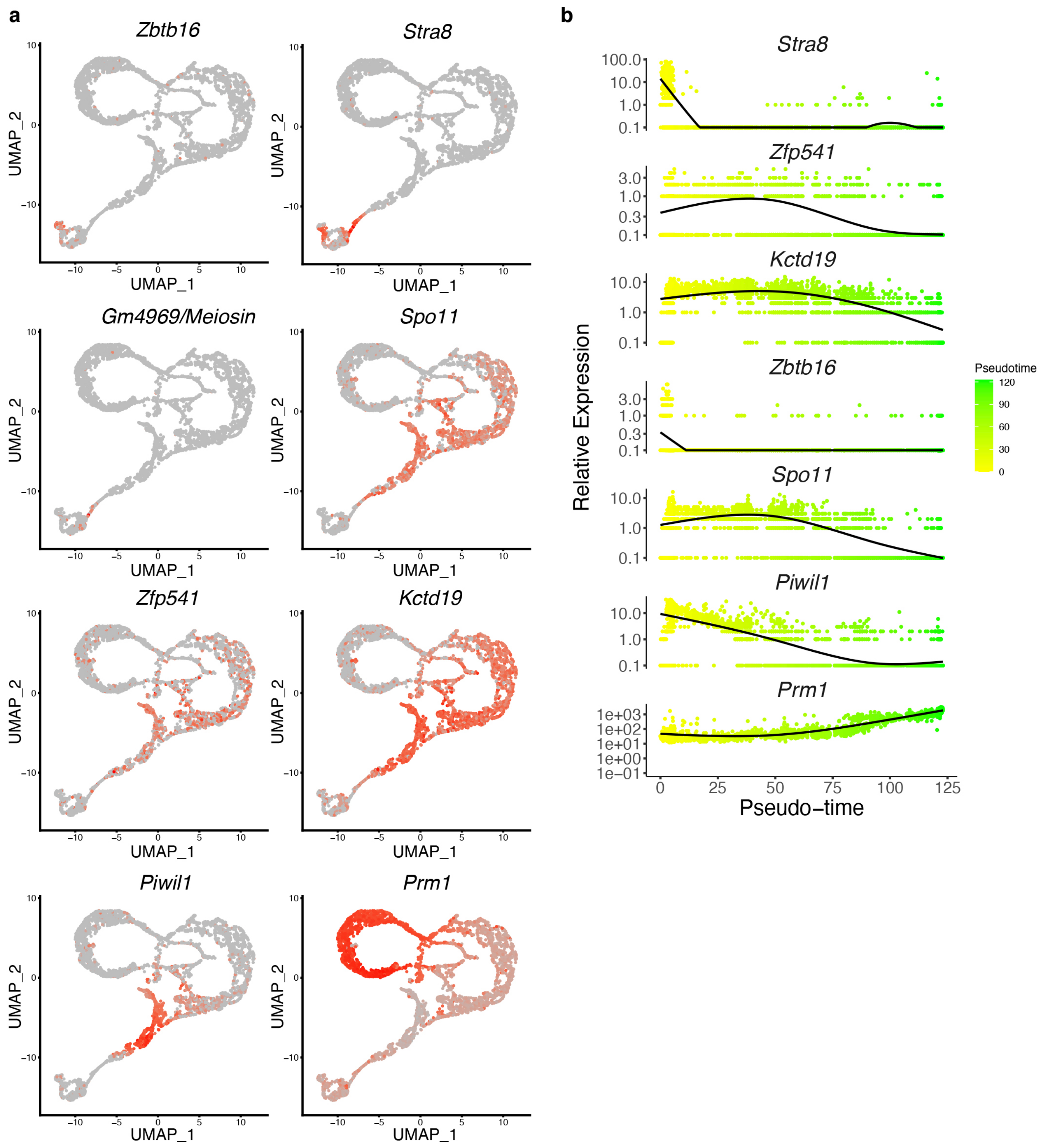

Supplementary Figure 1. Expression pattern of *Zfp541* and *Kctd19* during spermatogenesis

(a) UMAP plots show scRNA-seq profiling of spermatogenic cells from adult mouse testis. Expression patterns of *Zfp541*, *Kctd19* and other key developmental genes are presented on UMAP plots by reanalyzing scRNA-seq data (GEO : GSE109033) [1] of spermatogenic cells. Key developmental genes include *Zbtb16*: spermatogonia, *Stra8*: differentiating spermatogonia and preleptotene spermatocyte, *Gm4969/Meiosin*: preleptotene spermatocyte, *Spo11*: meiotic prophase spermatocyte, *PiwiL1*: spermatogonia and meiotic prophase spermatocyte, and *Prm1*: spermiogenesis.

(b) Expression patterns of *Zfp541*, *Kctd19* and other key developmental genes over pseudotime in adult mouse testis.

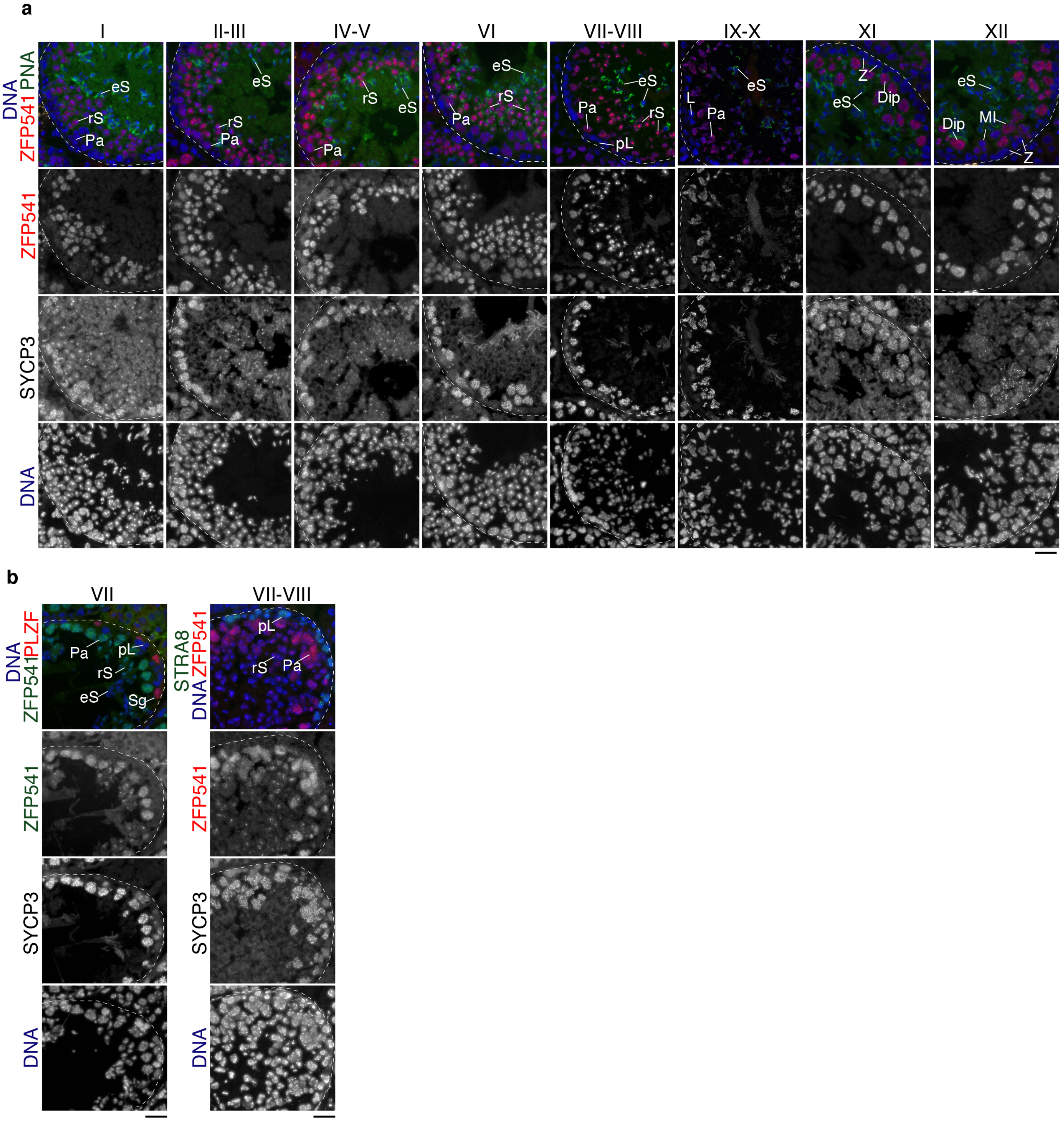

Supplementary Figure 2. Expression pattern of ZFP541 protein in seminiferous tubule cycles

(a) Seminiferous tubule sections from WT testis (8-weeks old) were stained as indicated.

(b) Stage VII seminiferous tubule sections from WT testis (8-weeks old) were stained as indicated.

Sg: Spermatogonia, pL: preleptotene L: leptotene, Z: zygotene, Pa: pachytene, Dip: diplotene, MI: metaphase I, rS: round spermatid, eS: elongated spermatid. Boundaries of the seminiferous tubules are indicated by white dashed lines. Roman numbers indicate the seminiferous tubule stages. Scale bars: 25 µm.

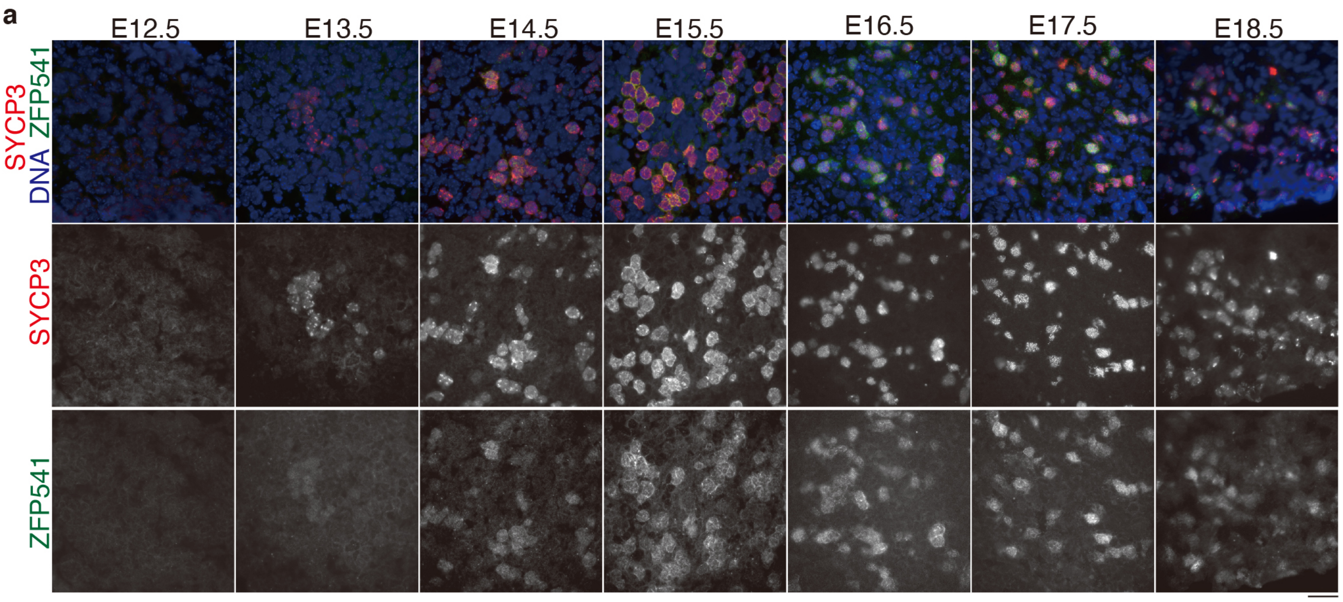

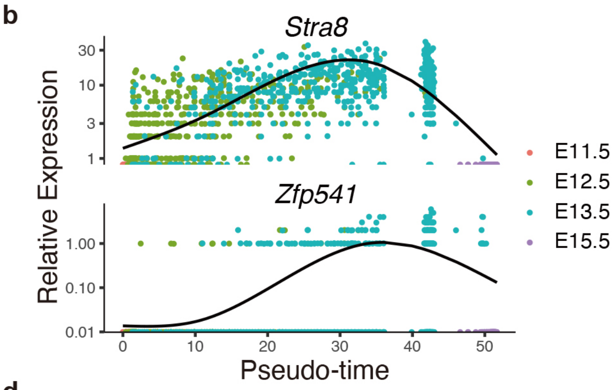

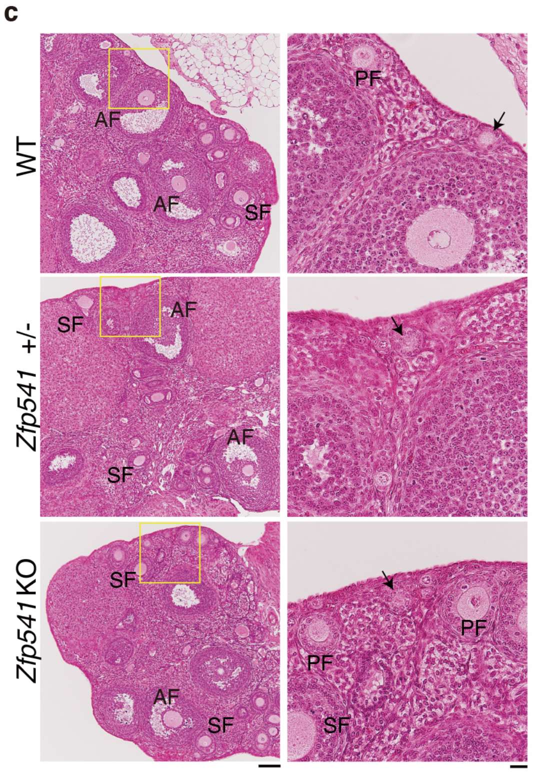

d

♀ x Hetero ♂	pups / mating duration
Hetero ♀ ID14	19 (5+8+6) for 24weeks
Hetero ♀ ID21	7 for 24weeks
Hetero ♀ ID44	20 (5+8+7) for 24weeks
Hetero ♀ ID47	12 (8+4) for 24weeks
Hetero ♀ ID9	10 (6+4) for 24weeks
Hetero ♀ ID10	20 (7+7+6) for 24weeks
KO ♀ ID20	11 (8+3) for 24weeks
KO ♀ ID41	17 (2+4+11) for 24weeks
KO ♀ ID45	17 (4+6+7) for 24weeks
KO ♀ ID8	5 for 24weeks

Supplementary Figure 3. Phenotypic analyses of *Zfp541* KO ovaries

(a) WT embryonic ovary sections were stained as indicated. Scale bar: 25 µm.

(b) Expression profiles of *Zfp541* and *Stra8* in E11.5, E12.5, E13.5, E15.5 fetal ovaries along pseudotime trajectory of germ cells. Pseudotime analysis was performed by reanalyzing scRNA-seq data (DRA011172) [2].

(c) Hematoxylin and eosin staining of the sections from WT, *Zfp541* +/- and *Zfp541* KO ovaries (8-weeks old). Scale bar: 100 µm. Enlarged images are shown on the right. Scale bar: 20 µm. Arrow: primordial follicle. PF: primary follicle, SF: secondary follicle, AF: antral follicle. Biologically independent mice (N=3) for each genotype were examined.

(d) Fertility of *Zfp541* KO females was examined by mating with *Zfp541* +/- males for the indicated period.

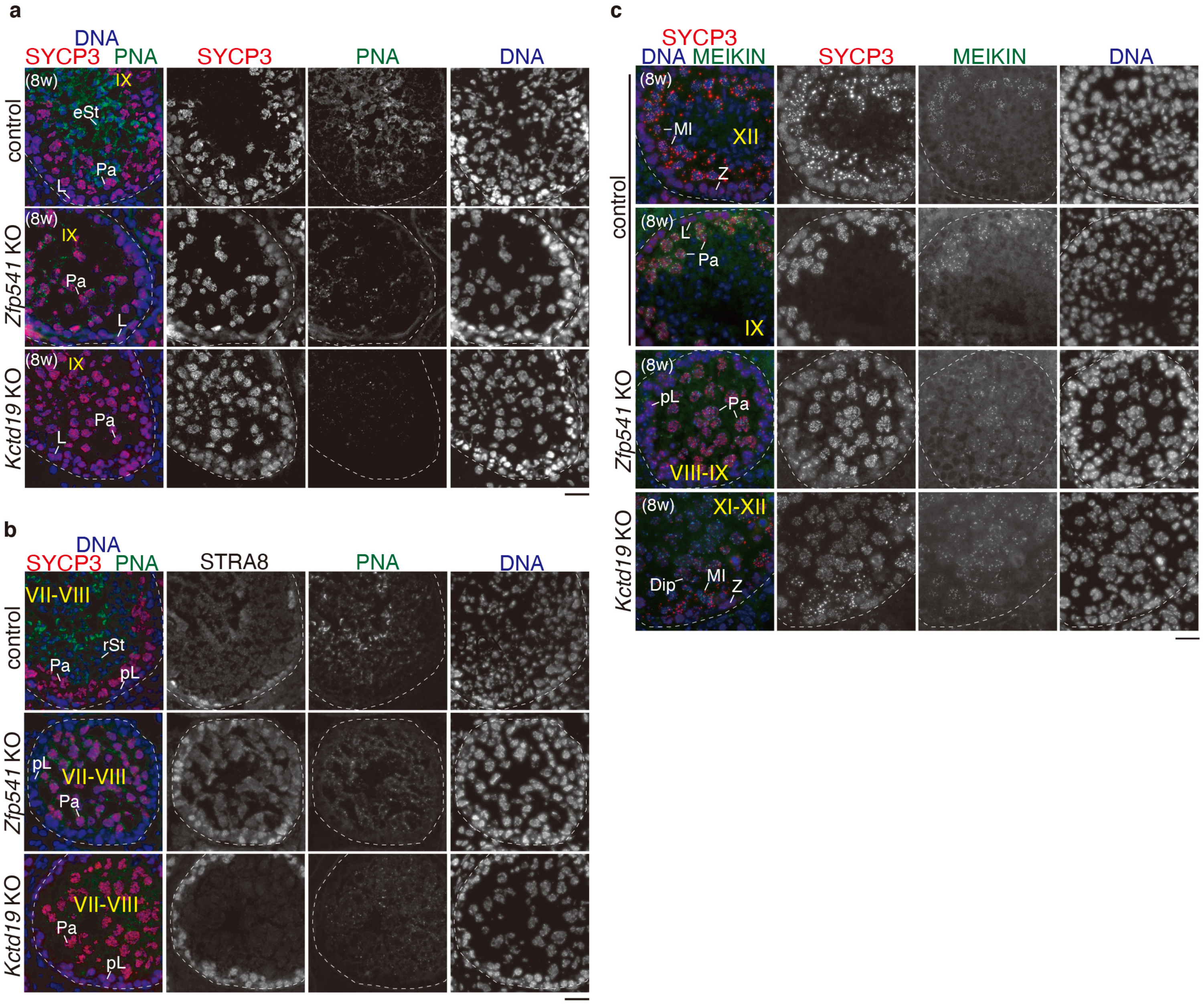

Supplementary Figure 4. Comparable immunostaining of PNA lectin and MEIKIN markers in *Zfp541* KO and *Kctd19* KO seminiferous tubules

(a) Seminiferous tubule sections (8-weeks old) were stained for SYCP3, PNA lectin and DAPI. Note that the seminiferous tubule that contained PNA-positive elongated spermatids were not identified in *Zfp541* KO and *Kctd19* KO testes.

(b) Seminiferous tubule sections (8-weeks old) were stained for SYCP3, PNA lectin, STRA8 and DAPI. STRA8 is a marker that detects stage VII-VIII tubules. Note that the seminiferous tubule that contained PNA-positive round or elongated spermatids were not identified in *Zfp541* KO and *Kctd19* KO testes.

(c) Seminiferous tubule sections (8-weeks old) were stained for SYCP3, MEIKIN and DAPI. MEIKIN localizes to the kinetochore from late pachytene to metaphase I [3]. Metaphase I spermatocytes were identified by MEIKIN+/ centromeric SYCP3 signals. Note that whereas stage XII tubules that contained metaphase I spermatocytes were identified in the control and *Kctd19* KO testes, any XII tubule was not found in *Zfp541* KO.

pL: preleptotene, L: leptotene, Z: zygotene, Pa: pachytene, Dip: diplotene. M I: Metaphase I, rSt: round spermatid, eSt: elongated spermatid. Boundaries of the seminiferous tubules are indicated by white dashed lines. Roman numbers indicate the seminiferous tubule stages. Scale bars: 25 µm.

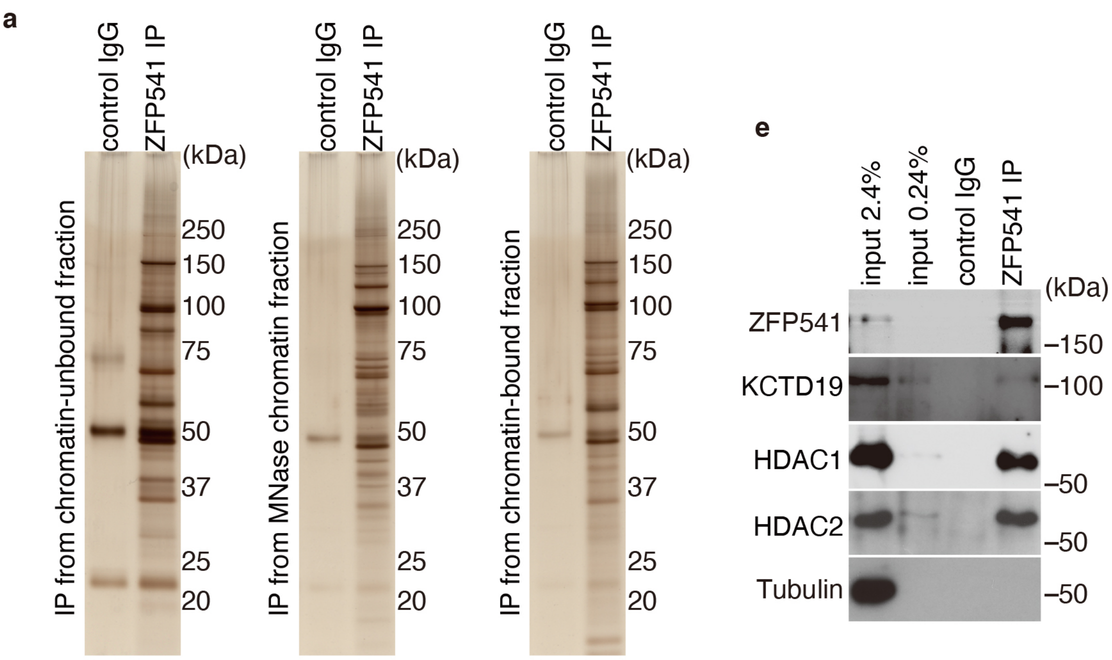

b

Zfp541 IP Chr unbound fraction IP	1 st		2nd	
Reference	Coverage	Peptide (Hits)	Coverage	Peptide (Hits)
**zinc finger protein 541**	38.60	71 (71 0 0 0 0)	45.90	94 (94 0 0 0 0)
**potassium channel tetramerisation domain containing 19**	45.60	68 (68 0 0 0 0)	52.20	91 (91 0 0 0 0)
**histone deacetylase 2**	25.40	54 (54 0 0 0 0)	34.00	55 (55 0 0 0 0)
HSP72_MOUSE Heat shock-related 70 kDa protein 2	38.40	45 (45 0 0 0 0)	40.60	41 (41 0 0 0 0)
**histone deacetylase 1**	21.80	44 (44 0 0 0 0)	28.20	42 (42 0 0 0 0)
**terminal deoxynucleotidyltransferase interacting factor 1**	39.00	20 (20 0 0 0 0)	39.00	19 (19 0 0 0 0)
tubulin, alpha 1a	20.00	14 (14 0 0 0 0)	16.70	12 (12 0 0 0 0)
MYH11_MOUSE Myosin-11 (Myosin heavy chain 11)	11.50	12 (12 0 0 0 0)	8.60	9 (9 0 0 0 0)
lactate dehydrogenase C	18.40	7 (7 0 0 0 0)	33.70	8 (8 0 0 0 0)
put. beta-actin (aa 27-375)	19.50	5 (5 0 0 0 0)	33.80	8 (8 0 0 0 0)

c

Zfp541 MNase Chromatin fraction IP	1 st		2nd	
Reference	Coverage	Peptide (Hits)	Coverage	Peptide (Hits)
matrin 3 [Mus musculus]	42.10	111 (111 0 0 0 0)	38.90	89 (89 0 0 0 0)
SFR14_MOUSE Putative splicing factor, arginine/serine-rich 14	35.10	39 (39 0 0 0 0)	41.80	44 (44 0 0 0 0)
**potassium channel tetramerisation domain containing 19**	25.80	21 (21 0 0 0 0)	33.40	24 (24 0 0 0 0)
**zinc finger protein 541**	22.40	19 (19 0 0 0 0)	31.00	25 (25 0 0 0 0)
tubulin, beta 5	46.60	14 (14 0 0 0 0)	47.10	14 (14 0 0 0 0)
heterogeneous nuclear ribonucleoprotein M isoform a	36.90	14 (14 0 0 0 0)	27.60	14 (14 0 0 0 0)
heterogeneous nuclear ribonucleoprotein H1	28.70	10 (10 0 0 0 0)	26.70	10 (10 0 0 0 0)
heterogeneous nuclear ribonucleoprotein F	23.60	8 (8 0 0 0 0)	30.80	10 (10 0 0 0 0)
**terminal deoxynucleotidyltransferase interacting factor 1**	36.60	7 (7 0 0 0 0)	29.90	6 (6 0 0 0 0)
interleukin enhancer binding factor 2	31.80	7 (7 0 0 0 0)	10.30	4 (4 0 0 0 0)
unnamed protein product	10.00	6 (6 0 0 0 0)	11.20	7 (7 0 0 0 0)
**histone deacetylase 2**	15.00	6 (6 0 0 0 0)	12.50	5 (5 0 0 0 0)
tubulin, alpha 1a	12.70	6 (6 0 0 0 0)	6.40	4 (4 0 0 0 0)
**histone deacetylase 1**	13.90	4 (4 0 0 0 0)	20.10	6 (6 0 0 0 0)
myelin basic protein expression factor 2, repressor	7.40	4 (4 0 0 0 0)	18.50	5 (5 0 0 0 0)
PREDICTED: similar to SMPTB	9.90	4 (4 0 0 0 0)	10.80	4 (4 0 0 0 0)
RNA binding motif protein 14	12.30	4 (4 0 0 0 0)	13.20	4 (4 0 0 0 0)

d

Zfp541 high salt Chromatin fraction IP	1 st		2nd	
Reference	Coverage	Peptide (Hits)	Coverage	Peptide (Hits)
**zinc finger protein 541**	38.00	42 (42 0 0 0 0)	38.70	31 (31 0 0 0 0)
**potassium channel tetramerisation domain containing 19**	40.40	38 (38 0 0 0 0)	47.60	39 (39 0 0 0 0)
matrin 3	36.20	39 (39 0 0 0 0)	37.10	39 (39 0 0 0 0)
tubulin, beta 5	47.10	15 (15 0 0 0 0)	36.00	12 (12 0 0 0 0)
SFR14_MOUSE Putative splicing factor, arginine/serine-rich 14	20.10	11 (11 0 0 0 0)	29.40	16 (16 0 0 0 0)
golgi autoantigen, golgin subfamily a, 5	25.20	9 (9 0 0 0 0)	17.30	8 (8 0 0 0 0)
**terminal deoxynucleotidyltransferase interacting factor 1**	39.00	9 (9 0 0 0 0)	33.50	8 (8 0 0 0 0)
**histone deacetylase 2**	12.50	8 (8 0 0 0 0)	16.20	7 (7 0 0 0 0)
heterogeneous nuclear ribonucleoprotein F	30.80	7 (7 0 0 0 0)	27.70	6 (6 0 0 0 0)
heterogeneous nuclear ribonucleoprotein M	14.70	7 (7 0 0 0 0)	23.50	11 (11 0 0 0 0)
**histone deacetylase 1**	20.10	6 (6 0 0 0 0)	19.90	9 (9 0 0 0 0)
tubulin, alpha 8	20.70	6 (6 0 0 0 0)	25.20	7 (7 0 0 0 0)
put. beta-actin (aa 27-375)	29.50	5 (5 0 0 0 0)	16.30	4 (4 0 0 0 0)
heterogeneous nuclear ribonucleoprotein H1	13.80	4 (4 0 0 0 0)	20.30	6 (6 0 0 0 0)

Supplementary Figure 5. MS analyses of ZFP541 interacting factors in testis extracts

(a) Silver staining of the immunoprecipitates by anti-ZFP541 antibody (ZFP541-IP) from the chromatin-unbound, MNase released chromatin, and chromatin-bound fractions of the testis extracts.

(b-d) The immunoprecipitates were subjected to liquid chromatography tandem-mass spectrometry (LC-MS/MS) analyses. The proteins identified by the LC-MS/MS analyses of ZFP541-IP are presented after excluding the proteins detected in the control IgG-IP. The proteins, which were reproducibly identified by two independent LC-MS/MS analyses (1st and 2nd) with more than 3 different peptide hits, are listed with the number of peptide hits and % coverage in the table. Shown are the identified proteins of ZFP541 immunoprecipitates from chromatin-unbound **(b)**, MNase released chromatin **(c)**, and chromatin-bound **(d)** fractions of the testis. The proteins reproducibly identified in the three different fractions are shown in bold.

(e) Immunoblot showing the immunoprecipitates of ZFP541 from chromatin extracts of WT mouse testes.

a

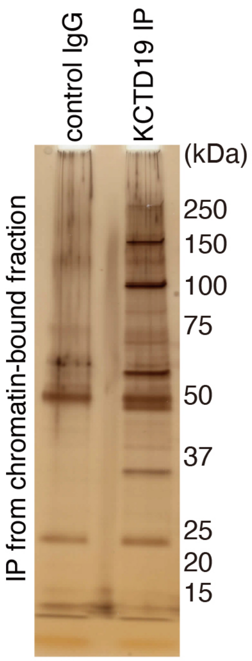

b

Description	KCTD19 IP (chr bound)	
	Score	PSMs
Zinc finger protein 541	3303.99	178
BTB/POZ domain-containing protein KCTD19	2648.31	171
Heat shock-related 70 kDa protein 2	1303.54	63
Histone deacetylase 1	1108.03	117
Deoxynucleotidyltransferase terminal-interacting protein 1	1082.13	57
Protein transport protein sec16	1049.79	82
Heat shock cognate 71 kDa protein	852.08	41
Kinesin-like protein KIF20A	566.14	35
Histone deacetylase 2	470.52	48
Vimentin	446.18	33
Glutamine-rich protein 2	440.58	33
Tubulin alpha-1A	420.18	26
Tubulin beta-4B	383.51	31
Tubulin alpha-3	372.33	25
Tubulin beta-5	366.90	33
Nucleoporin NUP35	342.29	22
Tcfl5 protein	298.13	26
Hnrnpm	222.64	21
Stress-70 protein	211.13	18
Hspa5	186.09	13
Matr3	176.44	13
RNA binding protein fox-1 homolog 2	170.44	10
Tubulin beta chain	137.44	10
Mideas	100.10	9
Protein MB21D2	90.68	9
General vesicular transport factor p115	89.78	7
Heat shock factor protein 5	89.49	14
Hnrnpu	83.37	6
Centrosomal protein of 72 kDa	80.11	7
Transcriptional-regulating factor 1	78.83	4
RuvB-like 1	76.35	5
Centromere protein V	75.37	4
Heterogeneous nuclear ribonucleoprotein H	75.31	4
Rbm14	65.66	6
PBX1b	64.34	7
Androgen receptor	63.31	11

Supplementary Figure 6. MS analyses of KCTD19 interacting factors in testis extracts

(a) Silver staining of the immunoprecipitates by anti-KCTD19 antibody (KCTD19-IP) from the chromatin-bound fraction of the testis extracts. This data was acquired from a single experiment.

(b) The immunoprecipitates from the chromatin-bound fraction of the testis extracts were subjected to liquid chromatography tandem-mass spectrometry (LC-MS/MS) analyses. The proteins identified by the LC-MS/MS analysis of KCTD19-IP are presented after excluding the proteins detected in the control IgG-IP. The proteins with more than 3 different peptide hits are listed with the number of peptide hits and Mascot scores. The proteins commonly identified in KCTD19-IP and ZFP541-IP from the chromatin-bound fraction of the testis extracts are shown in red.

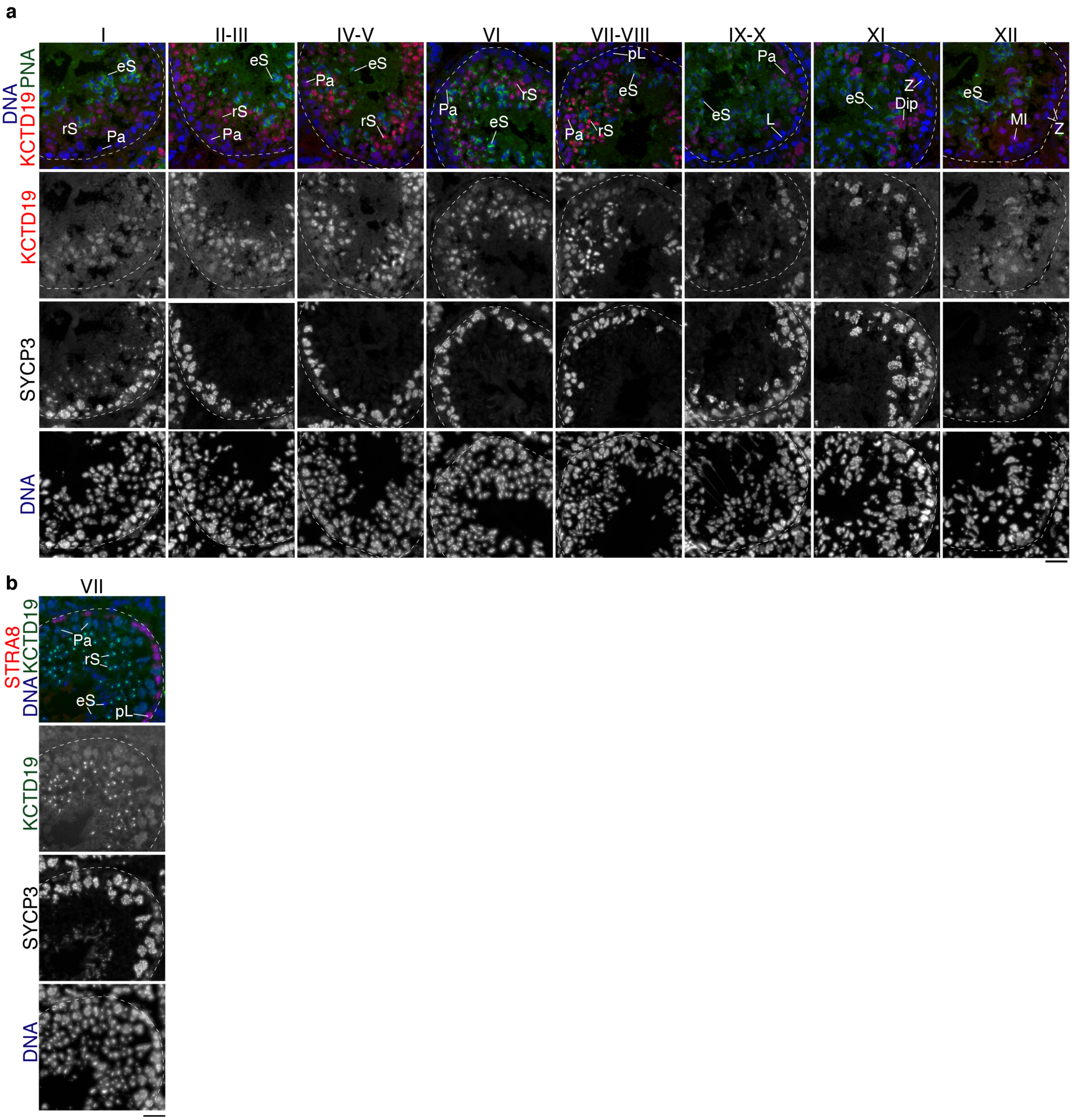

Supplementary Figure 7. Expression pattern of KCTD19 protein in seminiferous tubule cycles

(a) Seminiferous tubule sections from WT testis (8-weeks old) were stained as indicated.

(b) Stage VII seminiferous tubule sections from WT testis (8-weeks old) were stained as indicated.

pL: preleptotene L : leptotene, Z: zygotene, Pa: pachytene, Dip: diplotene, MI: metaphase I, rS: round spermatid, eS: elongated spermatid. Boundaries of the seminiferous tubules are indicated by white dashed lines. Roman numbers indicate the seminiferous tubule stages. Scale bars: 25 µm.

a

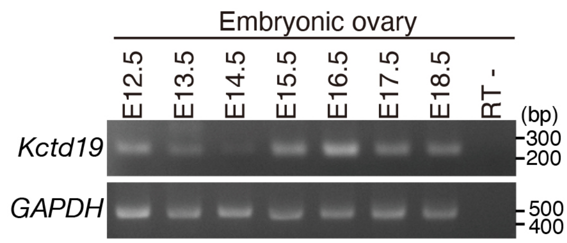

b

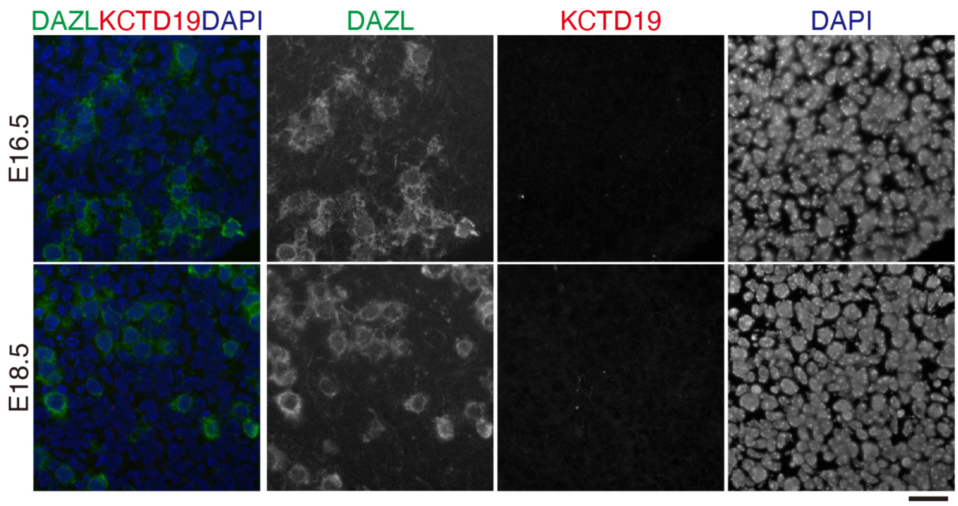

c

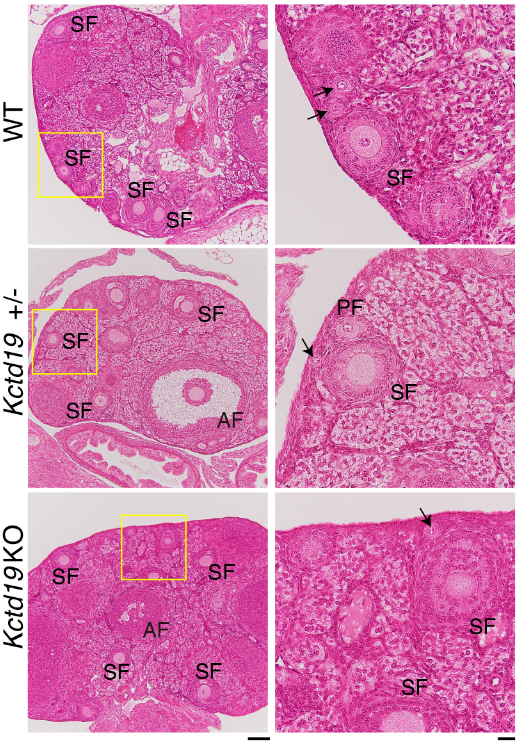

d

♀ x Hetero ♂	pups / mating duration
Hetero ♀ ID3	15 (7+8) for 25weeks
Hetero ♀ ID4	33 (5+11+9+8) for 25weeks
Hetero ♀ ID6	26 (10+8+8) for 25weeks
Hetero ♀ ID16	12 (7+5) for 25weeks
Hetero ♀ ID17	26 (7+4+7+8) for 25weeks
KO ♀ ID22	25 (8+10+7) for 25weeks
KO ♀ ID3	11 (5+6) for 25weeks
KO ♀ ID6	6 for 25weeks
KO ♀ ID8	8 for 25weeks
KO ♀ ID9	9 for 25weeks

Supplementary Figure 8. Phenotypic analyses of *Kctd19* KO ovaries

(a) RT-PCR analysis of *Kctd19* in embryonic ovaries (E12.5-18.5). RT- indicates control PCR without reverse transcription. The data was acquired from two separate experiments.

(b) Embryonic ovary sections (E16.5 and E18.5) were stained for KCTD19, DAPI and a germ cell marker DAZL. N=1 mouse from two separate experiments in E16.5, N=1 mouse from single experiment in E18.5. Scale bars: 25 µm.

(c) Hematoxylin and eosin staining of the sections from WT, *Kctd19* +/- and *Kctd19* KO ovaries (8-weeks old). Scale bar: 100 µm. Enlarged images are shown on the right. Scale bar: 20 µm. Arrow: primordial follicle, PF: primary follicle, SF: secondary follicle, AF: antral follicle. Biologically independent mice (N=3) for each genotype were examined.

(d) Fertility of *Kctd19* KO females was examined by mating with *Kctd19* +/- males for the indicated period.

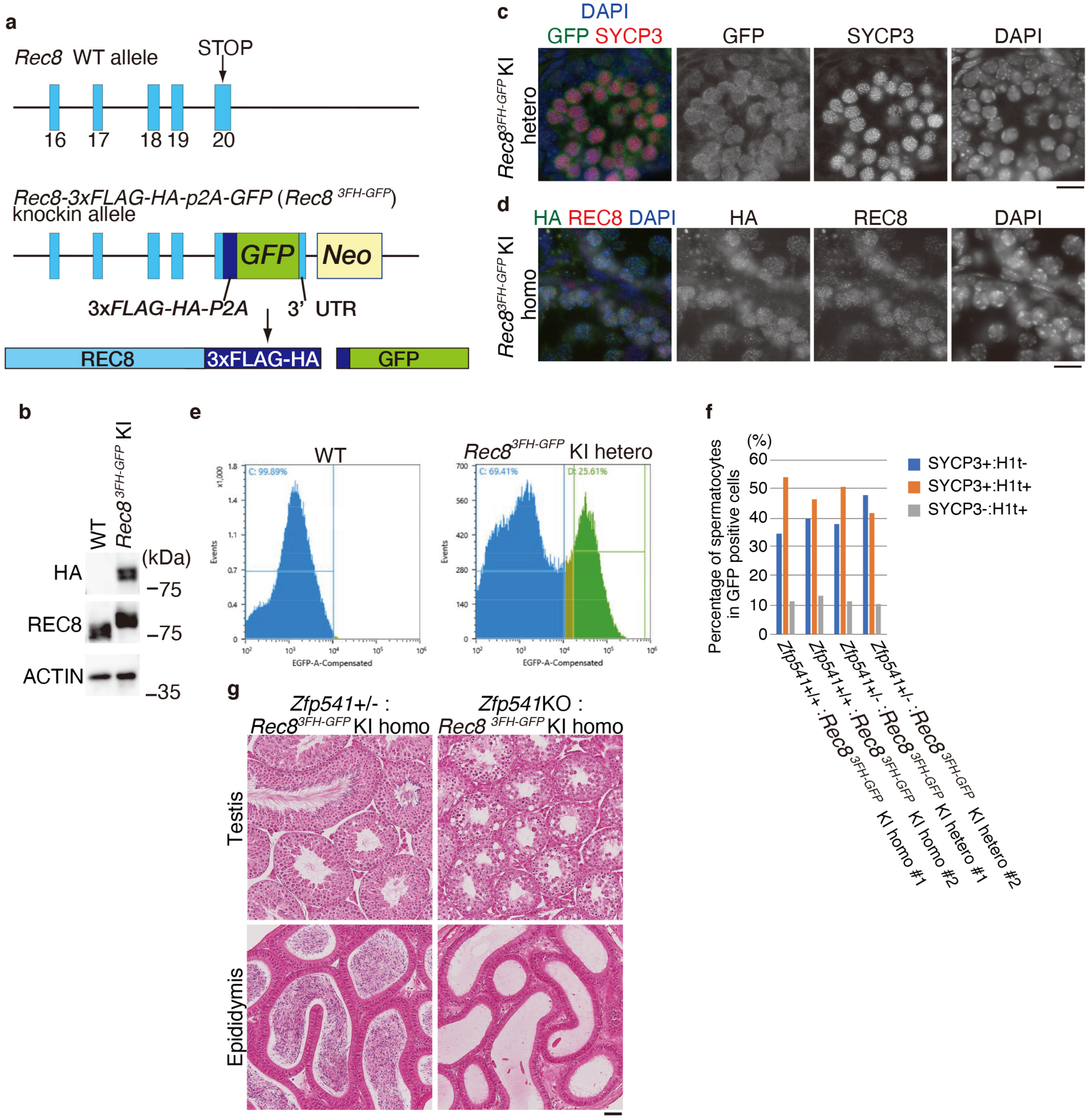

Supplementary Figure 9. Generation of *Rec8-3xFLAG-HA-p2A-GFP* knock-in mice

(a) Schematic illustrations of the *Rec8* WT allele and the *Rec8-3xFLAG-HA-p2A-GFP* knock-in (*Rec8-3FH-GFP* KI) allele. Blue boxes represent exons. Coding exon 20 is followed by *3xFLAG-HA-p2A-GFP* and the 3' UTR. Neo: Neomycine resistance gene.

(b) Immunoblot of testis extracts from WT (non-tagged control) and the *Rec8-3xFLAG-HA-p2A-GFP* KI homozygous mice. Note that anti-REC8 blot detected 3xFLAG-HA tagged REC8 at higher molecular weight than the endogenous REC8.

(c) Seminiferous tubule sections from *Rec8-3xFLAG-HA-p2A-GFP* KI heterozygous testis were stained for SYCP3, GFP and DAPI. Scale bar: 15 µm.

(d) Seminiferous tubule sections from *Rec8-3xFLAG-HA-p2A-GFP* KI homozygous testis were stained for HA, REC8 and DAPI. Scale bar: 15 µm.

(e) The meiotic prophase spermatocytes were isolated by fluorescent sorting of GFP positive cells from testes on the *Rec8-3FH-p2A-GFP* KI background.

(f) Cell population was counted for GFP positive spermatocytes isolated from *Rec8-3FH-GFP* KI background. Note that H1t positive cells were ~ 50% of GFP positive spermatocytes.

(g) Hematoxylin and eosin staining of the testes (upper) and epididymis (lower) sections from *Zfp541+/-* and *Zfp541* KO in the *Rec8-3HA-GFP* KI homozygous background (8-weeks old). Scale bar: 50 µm.

Note that the REC8-3xFLAG-HA fusion protein localizes along the axes in a similar manner to that observed in normal WT testis. The fusion protein was physiologically functional considering that homozygous male and female mice with the KI allele showed normal fertility.

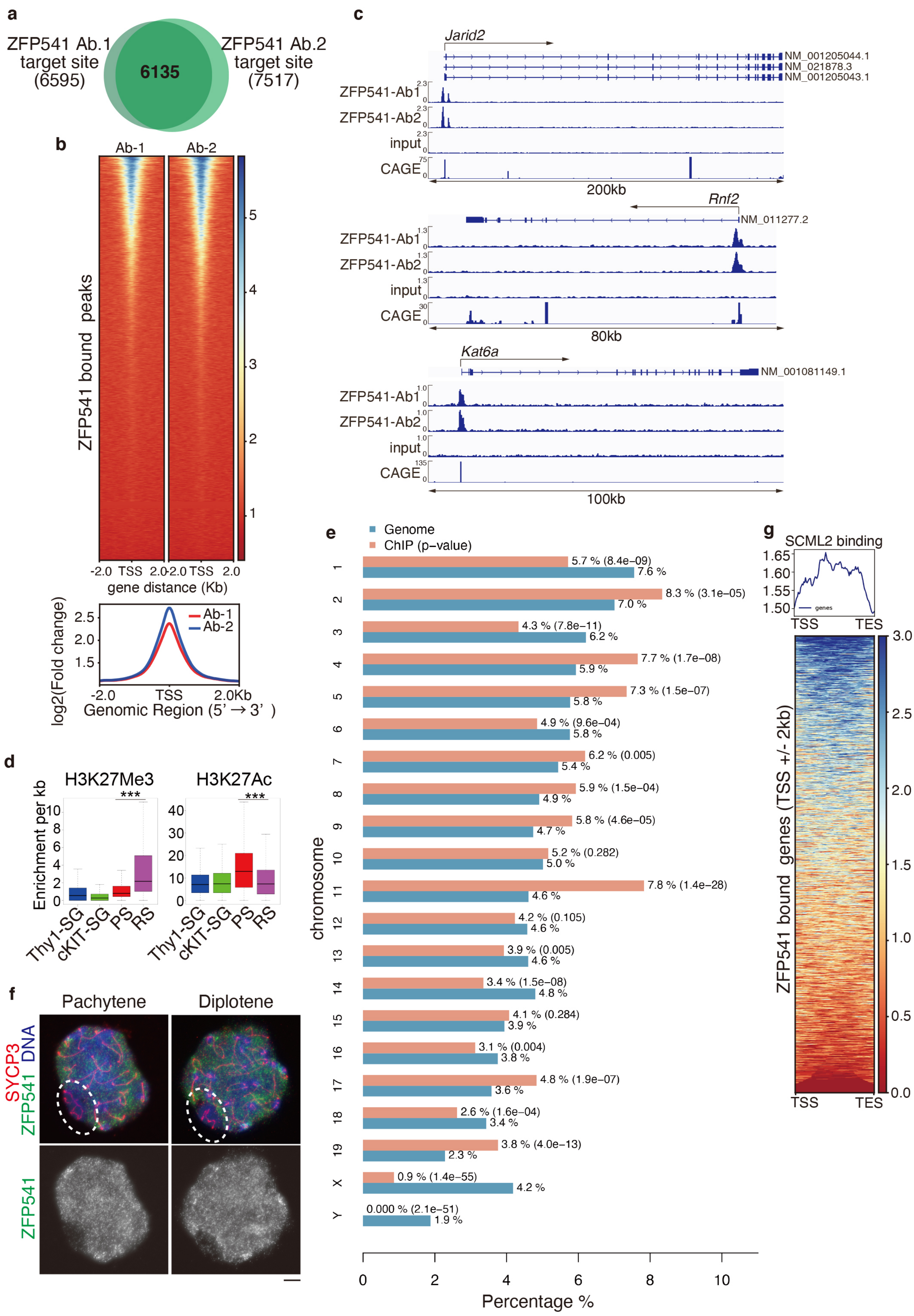

a
ZFP541 Ab.1 target site (6595)
6135
ZFP541 Ab.2 target site (7517)
b
Ab-1
Ab-2
ZFP541 bound peaks
-2.0 TSS 2.0 -2.0 TSS 2.0
gene distance (Kb)
log2(Fold change)
Ab-1
Ab-2
-2.0 TSS 2.0Kb
Genomic Region (5' → 3')
c
Jarid2
NM_001205044.1
NM_021878.3
NM_001205043.1
ZFP541-Ab1
ZFP541-Ab2
input
CAGE
200kb
Rnf2
NM_011277.2
80kb
Kat6a
NM_001081149.1
100kb
d
H3K27Me3
H3K27Ac
Enrichment per kb
Thy1-SG
cKIT-SG
PS
RS
e
Genome
ChIP (p-value)
chromosome
5.7 % (8.4e−09) 7.6 %
8.3 % (3.1e−05) 7.0 %
4.3 % (7.8e−11) 6.2 %
7.7 % (1.7e−08) 5.9 %
7.3 % (1.5e−07) 5.8 %
4.9 % (9.6e−04) 5.8 %
6.2 % (0.005) 5.4 %
5.9 % (1.5e−04) 4.9 %
5.8 % (4.6e−05) 4.7 %
5.2 % (0.282) 5.0 %
7.8 % (1.4e−28) 4.6 %
4.2 % (0.105) 4.6 %
3.9 % (0.005) 4.6 %
3.4 % (1.5e−08) 4.8 %
4.1 % (0.284) 3.9 %
3.1 % (0.004) 3.8 %
4.8 % (1.9e−07) 3.6 %
2.6 % (1.6e−04) 3.4 %
3.8 % (4.0e−13) 2.3 %
0.9 % (1.4e−55) 4.2 %
0.000 % (2.1e−51) 1.9 %
Percentage %
f
Pachytene
Diplotene
SYCP3
ZFP541
DNA
ZFP541
g
SCML2 binding
genes
TSS
TES
ZFP541 bound genes (TSS +/- 2kb)

Supplementary Figure 10. ChIP-seq analysis of ZFP541 in the testis

(a) Venn diagram representing the overlap of ZFP541-bound sites (6135 sites) from two independent ZFP541 ChIP-seq data sets using two different antibodies.

(b) Heat map of the common ZFP541 binding sites (6135 sites) of two independent ZFP541 ChIP-seq at the positions -2.0 kb upstream to +2.0 kb downstream relative to the TSS. Average distributions of ZFP541-binding peak for two independent ZFP541 ChIP-seq are shown on the bottom.

(c) Genomic view of ZFP541 ChIP-seq (duplicates using two different anti-ZFP541antibodies, Ab-1 and Ab-2) and input DNA data over representative gene loci. Genomic coordinates were obtained from RefSeq. To specify testis specific TSS, RNA-seq of the 5' capped end of the mRNA (CAGE) in P10.5 testis are shown (Li et al., 2013).

(d) Enrichment of H3K27me3 (left) and H3K27Ac (right) levels on the whole ZFP541-bound sites (6135 sites/5923 nearest genes) during spermatogenesis are shown by box-whisker plot (25th and 75th percentiles quantile with median : Whiskers indicate the minimum and max values. Bounds of box indicate lower and upper quartiles. The center bar indicates the median). Thy-SG: Thy+ spermatogonia, cKIT-SG: cKIT+ spermatogonia, PS: pachytene spermatocytes, RS: round spermatid. *** $p < 2.2 \times 10^{-16}$ (two sided Wilcoxon rank sum test). ChIP-seq data of H3K27me3 and H3K27Ac are derived from previous studies [4, 5]. N = 2 biologically independent samples.

(e) Distribution of ZFP541 ChIP-seq peaks on the chromosomes. Proportion (%) of the chromosome which were assigned with ZFP541 ChIP-seq peaks are shown with *p*-values. *p*-values are calculated using one-sided binomial test. Note that ZFP541 ChIP-seq peaks were excluded from the Y chromosome, and were less presented in the X chromosome.

(f) Pachytene and diplotene spermatocyte nuclei were immunostained for SYCP3 and ZFP541. The XY body is encircled by dashed lines. Independent germ cells (at least N=2) for each stage were examined in a single experiment. Scale bar: 5 µm.

(g) Heat map of SCML2 levels[6] on the ZFP541-target genes that are within +/- 2kb of TSS (4,689 genes). SCML2 levels are shown on the genomic regions between TSS and TES. Color key is shown. Average distributions of SCML2 are shown (upper).

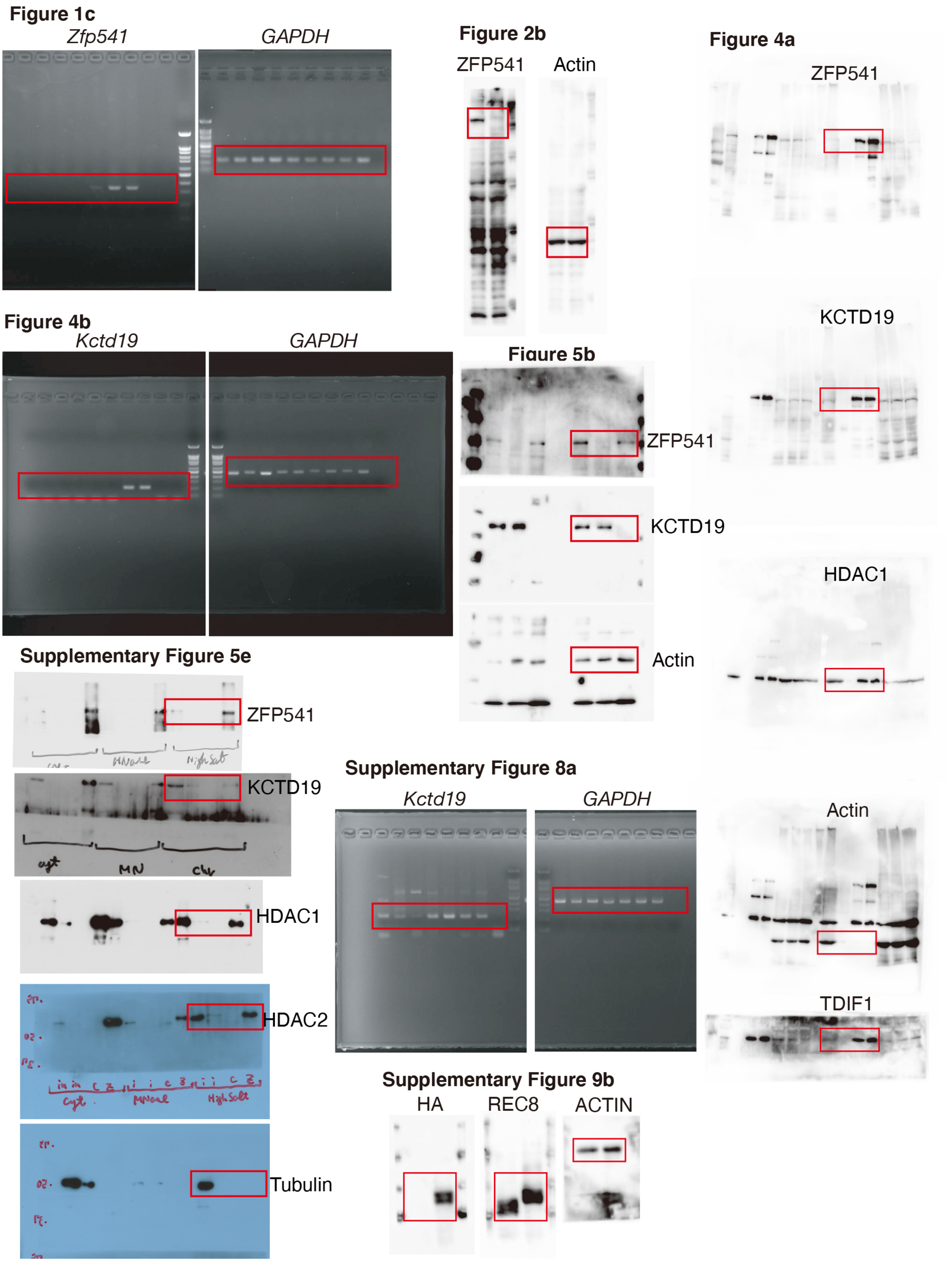

Supplementary Figure 11. Uncropped images of gels and blots

Full-length / uncropped images of agarose gel (Fig1c, Fig4b, Supplementary Fig8a) and immunoblots (Fig2b, Fig4a, Fig5b, Supplementary Fig5e, Supplementary Fig9b) are shown. For immunoblot of Fig2b, testis extracts were run on the same gel and blotted to the same membrane. Blotted membrane was sequentially reprobed with different antibodies. For immunoblot of Fig4a, the input testis extracts and immunoprecipitates were run on the same gel and the blotted membrane was sequentially reprobed with different antibodies. For TDIF1 immunoblot, the same membrane was stripped, cut according to molecular weight marker and reprobed with TDIF1 antibody. For Fig5b, Supplementary Fig5e and Supplementary Fig9b, the samples were run on the same gels and the blotted membranes were cut into two parts according to molecular weight marker, so that different proteins could be simultaneously probed with different antibodies. Those membranes were sequentially reprobed with different antibodies.

Supplementary references

1. Hermann, B. P. *et al*. The Mammalian Spermatogenesis Single-Cell Transcriptome, from Spermatogonial Stem Cells to Spermatids. *Cell Rep* **25**, 1650-1667 e1658, doi:10.1016/j.celrep.2018.10.026 (2018).
2. Shimada, R. *et al*. NANOS2 suppresses the cell cycle by repressing mTORC1 activators in embryonic male germ cells. *bioRxiv*, doi:doi.org/10.1101/2020.09.23.310912 (2020).
3. Kim, J. *et al*. Meikin is a conserved regulator of meiosis-I-specific kinetochore function. *Nature* **517**, 466-471, doi:10.1038/nature14097 (2015).
4. Maezawa, S. *et al*. Polycomb protein SCML2 facilitates H3K27me3 to establish bivalent domains in the male germline. *Proc Natl Acad Sci U S A* **115**, 4957-4962, doi:10.1073/pnas.1804512115 (2018).
5. Maezawa, S. *et al*. Super-enhancer switching drives a burst in gene expression at the mitosis-to-meiosis transition. *Nat Struct Mol Biol* **27**, 978-988, doi:10.1038/s41594-020-0488-3 (2020).
6. Hasegawa, K. *et al.* SCML2 establishes the male germline epigenome through regulation of histone H2A ubiquitination. *Dev Cell* **32**, 574-588, doi:10.1016/j.devcel.2015.01.014 (2015).
