# Supplementary material for: Meiosis-specific ZFP541 repressor complex promotes developmental progression of meiotic prophase towards completion during mouse spermatogenesis
Source: Nat Commun. 2021 Jun 1;12:3184. doi: 10.1038/s41467-021-23378-4 (PMC8169937; doi:10.1038/s41467-021-23378-4)
Supplement: Supplementary file 2 — Description of Additional Supplementary Files [file 41467_2021_23378_MOESM2_ESM.pdf]

Title: Supplementary Data 1.
Description: Differentially expressed genes in RNA-seq of WT vs Zfp541KO. The complete list of upregulated- and downregulatedgenes in Zfp541 KO, and their GO terms are shown.

Title: Supplementary Data 2.
Description: The complete gene list of the GO analyses for the clusters of DEGs in scRNA-seq. In each tub, shown are the complete list of GO terms for the clusters in upregulated- and downregulated-genes in scRNA-seq.

Title: Supplementary Data 3.
Description: The complete gene list of ZFP541-ChIP targets and their GO analyses. In each tub, shown are the complete list of ZFP541-ChIP nearest genes, ZFP541-binding sites resided within +/− 2 kb of the TSS (4,689 genes), those ZFP541-targets that were upregulated or downregulated in Zfp541 KO, their GO terms.

Title: Supplementary Data 4.
Description: The complete gene list of the GO analyses for the clusters of the ZFP541-target upregulated genes in scRNA-seq. In each tub, shown are the complete list of GO terms for the clusters in ZFP541-target upregulated genes in scRNA-seq.

Title: Supplementary Data 5.
Description: Primers and oligos used in this study
