# Supplementary material for: Meiosis-specific ZFP541 repressor complex promotes developmental progression of meiotic prophase towards completion during mouse spermatogenesis
Source: Nat Commun. 2021 Jun 1;12:3184. doi: 10.1038/s41467-021-23378-4 (PMC8169937; doi:10.1038/s41467-021-23378-4)
Supplement: Supplementary file 3 — Reporting Summary [file 41467_2021_23378_MOESM3_ESM.pdf]

nature research

Corresponding author(s): Kei-ichiro Ishiguro

Last updated by author(s): Apr 23, 2021

Reporting Summary

Nature Research wishes to improve the reproducibility of the work that we publish. This form provides structure for consistency and transparency in reporting. For further information on Nature Research policies, see our Editorial Policies and the Editorial Policy Checklist.

Statistics

For all statistical analyses, confirm that the following items are present in the figure legend, table legend, main text, or Methods section.

n/a	Confirmed	
☐	☒	The exact sample size (n) for each experimental group/condition, given as a discrete number and unit of measurement
☐	☒	A statement on whether measurements were taken from distinct samples or whether the same sample was measured repeatedly
☐	☒	The statistical test(s) used AND whether they are one- or two-sided<br>*Only common tests should be described solely by name; describe more complex techniques in the Methods section.*
☐	☒	A description of all covariates tested
☐	☒	A description of any assumptions or corrections, such as tests of normality and adjustment for multiple comparisons
☐	☒	A full description of the statistical parameters including central tendency (e.g. means) or other basic estimates (e.g. regression coefficient) AND variation (e.g. standard deviation) or associated estimates of uncertainty (e.g. confidence intervals)
☐	☒	For null hypothesis testing, the test statistic (e.g. F, t, r) with confidence intervals, effect sizes, degrees of freedom and P value noted<br>*Give P values as exact values whenever suitable.*
☒	☐	For Bayesian analysis, information on the choice of priors and Markov chain Monte Carlo settings
☒	☐	For hierarchical and complex designs, identification of the appropriate level for tests and full reporting of outcomes
☒	☐	Estimates of effect sizes (e.g. Cohen's d, Pearson's r), indicating how they were calculated

Our web collection on statistics for biologists contains articles on many of the points above.

Software and code

Policy information about availability of computer code

Data collection	no software was used for the data collection.
Data analysis	GraphPad Prism8 (version 8.4.3), Microsoft Excel (version 16.48), SoftWoRx (ver.7.2.1, GE Healthcare)., Seurat packge for R (v.3.1.3), monocle (ver.2.14.0), R (ver. 3.6.2) , RStudio (ver.1.2.1335), Bioworks (Ver. 3.3; Thermo Scientific), Xcalibur (Version 4.0, Thermo Fisher Scientific)

For manuscripts utilizing custom algorithms or software that are central to the research but not yet described in published literature, software must be made available to editors and reviewers. We strongly encourage code deposition in a community repository (e.g. GitHub). See the Nature Research guidelines for submitting code & software for further information.

Data

Policy information about availability of data

All manuscripts must include a data availability statement. This statement should provide the following information, where applicable:

- Accession codes, unique identifiers, or web links for publicly available datasets
- A list of figures that have associated raw data
- A description of any restrictions on data availability

All data supporting the conclusions are present in the paper and the supplementary materials. A reporting summary for this Article is available as Supplementary Information file. The source data (for Fig.1b, Fig.1d, Fig.2c, Fig. 3a, Fig. 3b, Fig. 3c, Fig. 3d, Fig. 3e, Fig.3f, Fig. 5d, Fig. 6a, Fig. 6b, Fig. 6c, Fig. 6e, Fig. 6f, Fig. 6g, supplementary Fig. 9f) are provided with this paper. The original images for all of the figures in this paper are deposited in public depository http://dx.doi.org/10.17632/kxb38h7snx.1. The ZFP541 ChIP-seq data of mouse testes are deposited in the GEO Sequence Read Archive (SRA) under accession number GSE163916. RNA-seq data of the GFP positive cells from the control and Zfp541 KO are deposited under GSE163917.
ChIP-seq data of H3K27me324 and H3K27ac14 were derived from GSE89502 and GSE130652, respectively. Data for RNA-seq (THY1+SG, PS, RS)22 and RNA-seq (KIT +SG)24 were derived from GSE55060 and GSE89502, respectively. CAGE data were derived from GEO: GSE4469038. The scRNA-seq data of fetal ovaries was derived

from DRA 01117231. 10xGenomics Drop-seq data of mouse adult testis was derived from GEO: GSE10903329. Uncropped blots can be found in Supplementary Fig 11.

Field-specific reporting

Please select the one below that is the best fit for your research. If you are not sure, read the appropriate sections before making your selection.

- [x] Life sciences
- [ ] Behavioural & social sciences
- [ ] Ecological, evolutionary & environmental sciences

For a reference copy of the document with all sections, see nature.com/documents/nr-reporting-summary-flat.pdf

Life sciences study design

All studies must disclose on these points even when the disclosure is negative.

Sample size	No statistical method was used to predetermine sample size. We followed the conventional way of quantification accepted in many of the published paper in meiosis research field and determined the sample size according to published papers (Cobb, J., Cargile, B. & Handel, M. A. (1999) doi:10.1006/dbio.1998.9101, Maezawa, S. et al. (2020) doi:10.1038/s41594-020-0488-3, Ishiguro, K. I. et al. (2020) doi:10.1016/j.devcel.2020.01.010, Larose, H. et al., (2020) doi:10.1091/mbc.E20-05-0334.).
Data exclusions	No data was excluded.
Replication	Each conclusion in the manuscript was based on results that were reproduced in at least two independent experiments and in at least three independent mice of each genotype.
Randomization	Mice were categorized based on their genotypes. The genotypes were determined by PCR. For experiments other than those involving mice, samples were non-randomly chosen according to the genotype.
Blinding	The investigators were not blinded to allocation during the experiments or to outcome assessment.<br>This is because the phenotypes were quite obvious that observer can be sure without blind test.<br>Further, the observer unbiasedly and carefully performed the quantification with enough sample number to make sure the conclusion.

Reporting for specific materials, systems and methods

We require information from authors about some types of materials, experimental systems and methods used in many studies. Here, indicate whether each material, system or method listed is relevant to your study. If you are not sure if a list item applies to your research, read the appropriate section before selecting a response.

Materials & experimental systems

n/a	Involved in the study	
[ ]	[x]	Antibodies
[x]	[ ]	Eukaryotic cell lines
[x]	[ ]	Palaeontology and archaeology
[ ]	[x]	Animals and other organisms
[x]	[ ]	Human research participants
[x]	[ ]	Clinical data
[x]	[ ]	Dual use research of concern

Methods

n/a	Involved in the study	
[ ]	[x]	ChIP-seq
[x]	[ ]	Flow cytometry
[x]	[ ]	MRI-based neuroimaging

Antibodies

Antibodies used	The following antibodies were used for immunoblot (IB) and immunofluorescence (IF) studies: rabbit anti-Actin (IB, 1:2000, sigma A2066), rabbit anti-SYCP1 (IF, 1:1000, Abcam ab15090), mouse anti-H2AX (IF, 1:1000, Abcam ab26350), rabbit anti-H2AX (IF, 1:1000, Abcam ab11174), rabbit anti-H3S10p (IF, 1:2000, ab5176), rabbit anti-TDIF1 (IB, 1:1000, ab228703), rabbit anti-HDAC2 (IB, 1:1000, Abcam ab32117), mouse anti-HDAC1 (1:1000, Upstate 05-614), rabbit TDIF1 (IB, 1:1000, Abcam ab228703), guinea pig anti-H1t (IF, 1:2000, kindly provided by Marry Ann Handel) 7,-tubulin DM1A (IB, 1:2000, Sigma 05-829), rabbit anti-DAZL (IF, 1:1000, ab34139), mouse anti-SYCP1 (IF, 1:1000, our home made) 37, rat anti-SYCP3 (IF, 1:1000, our home made)26, gunia pig anti-SYCP3 (IF, 1:2000, our home made) 26, mouse anti-SYCP3 (our home made) 37, rabbit anti-MEIKIN (our home made) 32 (IF, 1:1000), rat anti-STRA8(IF, 1:1000, our home made)26, rabbit ZFP541-N (IF, IB, 1:1000), rabbit KCTD19-N (IB, 1:1000, our home made in this study), rat KCTD19-N (IF, 1:1000, our home made in this study), rabbit KCTD19-C (IF, 1:1000, our home made in this study), and rat KCTD19-C (IF, 1:1000, our home made in this study).<br>Following secondary antibodies were used : Goat anti-rat IgG-Alexa Fluour 647 (IF, 1:1000, Thermo Fisher, A21247), Goat anti-rabbit IgG-Alexa Fluour 647 (IF, 1:1000, Thermo Fisher, A21244), Donkey anti-mouse IgG-Alexa Fluour 647 (IF, 1:1000, Thermo Fisher, A31571), Donkey anti-rabbit IgG-Alexa Fluour 647 (IF, 1:1000, Thermo Fisher, A31573), Donkey anti-rabbit IgG-Alexa Fluour 555 (IF, 1:1000, Thermo Fisher, A31572), Donkey anti-rat IgG-Alexa Fluour 555 (IF, 1:1000, Thermo Fisher, A48270), Donkey anti-rabbit IgG-Alexa Fluour 568 (IF, 1:1000, Thermo Fisher, A10042), Goat anti-rat IgG-Alexa Fluour 568 (IF, 1:1000, Thermo Fisher, A11077), Goat

	anti-rabbit IgG-Alexa Fluour 568 (IF, 1:1000, Thermo Fisher, A11011), Goat anti-mouse IgG-Alexa Fluour 568 (IF, 1:1000, Thermo Fisher, A11004), Donkey anti-rabbit IgG-Alexa Fluour 488 (IF, 1:1000, Thermo Fisher, A21206), Donkey anti-rat IgG-Alexa Fluour 488 (IF, 1:1000, Thermo Fisher, A21208), Goat anti-Gunia pig IgG-Alexa Fluour 488 (IF, 1:1000, Abcam ab150185), Goat anti-Gunia pig IgG-Alexa Fluour 555 (IF, 1:1000, Abcam ab150186), Goat anti-Gunia pig IgG-Alexa Fluour 647 (IF, 1:1000, Abcam ab150187).
Validation	The newly generated antibodies in this study were validated by western blotting and immunostaining using WT and knockout mouse controls.<br>The following our home made antibodies were validated for immunofluorescence (IF) in our previous studies: mouse anti-SYCP1 (IF, 1:1000, our home made) 37, rat anti-SYCP3 (IF, 1:1000, our home made)26, gunia pig anti-SYCP3 (IF, 1:2000, our home made) 26, mouse anti-SYCP3 (our home made) 37, rabbit anti-MEIKIN (our home made) 32 (IF, 1:1000), rat anti-STRA8(IF, 1:1000, our home made)26.<br>The following antibodies were validated for immunoblot (IB) in manufacture's website :<br>rabbit anti-Actin (IB, 1:2000, sigma A2066), abbit anti-TDIF1 (IB, 1:1000, ab228703), rabbit anti-HDAC2 (IB, 1:1000, Abcam ab32117), mouse anti-HDAC1 (1:1000, Upstate 05-614), a-tubulin DM1A (IB, 1:2000, Sigma 05-829),<br>The following antibodies were validated for immunofluorescence (IF) in manufacture's website :, rabbit anti-SYCP1 (IF, 1:1000, Abcam ab15090), mouse anti-gH2AX (IF, 1:1000, Abcam ab26350), rabbit anti-gH2AX (IF, 1:1000, Abcam ab11174), rabbit anti-H3S10p (IF, 1:2000, ab5176), , rabbit TDIF1 (IB, 1:1000, Abcam ab228703), guinea pig anti-H1t (IF, 1:2000, kindly provided by Marry Ann Handel) 7, rabbit anti-DAZL (IF, 1:1000, ab34139).

Animals and other organisms

Policy information about studies involving animals; ARRIVE guidelines recommended for reporting animal research

Laboratory animals	Zfp541 and Kctd19 knockout mice and Rec8-3xFLAG-HA-p2A-GFP knock-in mouse lines were generated in this study.Zfp541 and Kctd19 knockout mice were C57BL/6 background. Rec8-3xFLAG-HA-p2A-GFP knock-in mice were congenic with the C57BL/6 background. Whenever possible, each knockout animal was compared to littermates or age-matched non-littermates from the same colony, unless otherwise described. We used 3w and 8w animals for histological analysis and expression analysis, 18-21 day juvenile mice for RNA-seq and ChIP-seq and chromosome spread IF experiments, otherwise indicated in the figure legends. Both males and females were used for Zfp541 and Kctd19 knockout mice. Males and females were used for Rec8-3xFLAG-HA-p2A-GFP knock-in mice.<br>Housing conditions for the mice were under 12 hours dark/12 hours light cycle, ambient temperature at 20-23 degree C and humidity 40-60 % .
Wild animals	No wild animal was used.
Field-collected samples	No field-collected samples were used in the study.
Ethics oversight	Animal experiments were approved by the Institutional Animal Ethics Committees of Kumamoto University (approval F28-078, A2020-006, A30-001, A28-026).

Note that full information on the approval of the study protocol must also be provided in the manuscript.

ChIP-seq

Data deposition

- [x] Confirm that both raw and final processed data have been deposited in a public database such as GEO.
- [x] Confirm that you have deposited or provided access to graph files (e.g. BED files) for the called peaks.

Data access links<br>*May remain private before publication.*	The ZFP541 ChIP-seq data of mouse testes are deposited in the GEO Sequence Read Archive (SRA) under accession number GSE163916. RNA-seq data is deposited under GSE163917.
Files in database submission	PROCESSED DATA FILES<br>WTtestes_Input.bw (bigwig)<br>WTtestes_ZFP541_ChIP_Antibody1.bw<br>WTtestes_ZFP541_ChIP_Antibody2.bw<br><br>RAW FILES (fastq)<br>WTtestes_input.fastq.gz<br>WTtestes_ZFP541_ChIP_Antibody1.fastq.gz<br>WTtestes_ZFP541_ChIP_Antibody2.fastq.gz
Genome browser session<br>(e.g. UCSC)	UCSC mm10

Methodology

Replicates	ZFP541 ChIP-seq replicates were performed with two different anti-ZFP541-N antibodies (#1 and #2).
Sequencing depth	GSM4990661 WTtestes_input 43176224-50bp, Single-end<br>GSM4990662 WTtestes_ZFP541_ChIP_Antibody1 35813716-50bp, Single-end

	GSM4990663 WTtestes_ZFP541_ChIP_Antibody2 34065854-50bp, Single-end
Antibodies	rabbit anti-ZFP541-#1, rabbit anti-ZFP541-#2
Peak calling parameters	Peak calling was performed using MACS program v2.1.0 (Zhang et al., 2008) (https://github.com/macs3-project/MACS) with the option (-g mm -p 0.00001).
Data quality	FastQC (version 0.11.17) was used for initial quality control of the reads.<br>6595 peaks in GSM4990662 (WTtestes_ZFP541_ChIP_Antibody1) and 7517 peaks in GSM4990663 (WTtestes_ZFP541_ChIP_Antibody2) were identified with p<0.00001.
Software	Bowtie2 v2.3.4.1, MACS program v2.1.0 (Zhang et al., 2008) (https://github.com/macs3-project/MACS) for Peak calling, bedtools program (v2.27.1) (Quinlan and Hall, 2010) (https://bedtools.readthedocs.io/en/latest/)., Cis-regulatory Element System (CEAS) v0.9.9.7 (package version 1.0.2), MEME-ChIP v5.1.1 website (http://meme-suite.org/tools/meme-chip) (Bailey, 2011) for Motif identification, deepTools (v3.1.0) for generation of BigWig files, Integrative Genomics Viewer software (v.2.8.3) http://software.broadinstitute.org/software/igv/home. , DAVID Bioinformatics Resources 6.8 (Huang da et al., 2009) (https://david.ncifcrf.gov/), deeptools program v3.5.0. for aggregation plots and heatmaps, GREAT website (v4.0.4) (http://great.stanford.edu/public/html/), bedtools program (v2.27.1)
